# An isolated pulmonary nodule secondary to *Streptococcus intermedius* infection in an otherwise healthy 10-year-old boy: A case report and literature review

**DOI:** 10.3389/fped.2022.921258

**Published:** 2022-09-07

**Authors:** Meixia Huang, Shuxian Li, Xiling Wu, Dan Xu, Lanfang Tang, Zhimin Chen

**Affiliations:** Department of Pulmonology, The Children's Hospital, Zhejiang University School of Medicine, National Clinical Research Center for Child Health, Hangzhou, China

**Keywords:** *Streptococcus intermedius*, infection, pulmonary nodule, metagenomics next-generation sequencing, ultrasonography-guided lung biopsy, case report

## Abstract

*Streptococcus intermedius*, as a Gram-positive commensal bacterium, tends to cause various infections, such as brain and liver abscesses, endocarditis, and empyema, especially in immunocompromised patients. However, an isolated pulmonary nodule caused by *S. intermedius* in previously healthy individuals without traditional risk factors is rarely reported. Herein, we present a case of a 10-year-old immunocompetent boy referred to our department with a 5-day history of intermittent, left-sided chest pain. Chest X-ray and computed tomography revealed a left lung nodule. Although his blood, sputum, and bronchoalveolar lavage fluid cultures were negative, metagenomic next-generation sequencing (mNGS) showed only the presence of *S. intermedius* in ultrasonography-guided lung biopsy tissue and pleural fluid (416 and 110 reads, respectively). He was then successfully treated with appropriate intravenous antibiotics and avoided surgical intervention. To the best of our knowledge, this is the first report of *S. intermedius*-related pulmonary nodule confirmed by mNGS analysis in healthy children. For achieving proper diagnosis and treatment, infection with *S. intermedius* should be included in the differential diagnosis when coming across such a similar pulmonary nodule. mNGS, as a valuable supplement to conventional culture methods, is an essential diagnostic tool for identifying pathogens without typical characteristics.

## Introduction

Solitary pulmonary nodules are rare in children. Identifying the etiology of pulmonary nodules can present a challenge to clinicians owing to the diversity of pathologies, such as hamartochondroma, *Cryptococcus neoformans*, abscesses, vasculitides, malignancy, and granulomatous diseases (1, 2). Consequently, a conscientious and fastidious diagnosis is essential to avoid inappropriate treatment.

*Streptococcus intermedius* (*S. intermedius*), as a member of the *Streptococcus anginosus* group (SAG), is a β-hemolytic Gram-positive microaerophilic coccus (3). Despite being a normal flora of the oral cavity, gastrointestinal, respiratory, and female urogenital tracts, *S. intermedius* is notorious for abscess formation, particularly in the brain (3). Underlying risk factors for *S. intermedius* infection include sinusitis, congenital heart disease, dental illness, oral manipulation, undergoing surgery, and chronic obstructive pulmonary disease (3). It is noteworthy that *S. intermedius* infection was more frequently identified in older patients with predisposing conditions (3). Still, healthy individuals typically do not develop an invasive infection with *S. intermedius*. Additionally, *S. intermedius* is most frequently associated with head and neck infections in children (4). Until now, there have been very few reported cases of *S. intermedius* causing isolated pulmonary nodules in the literature review (5), especially in children.

Herein, we describe a 10-year-old immunocompetent boy with a pulmonary nodule, which was ultimately confirmed by analysis of lung biopsy tissue with metagenomic next-generation sequencing (mNGS). Our case is extremely rare, as our patient is a young child without possible risk factors and he initially did not report fever, cough, or any other symptoms that may indicate lung infection and represented a diagnostic dilemma.

## Case presentation

**Figure 1:**
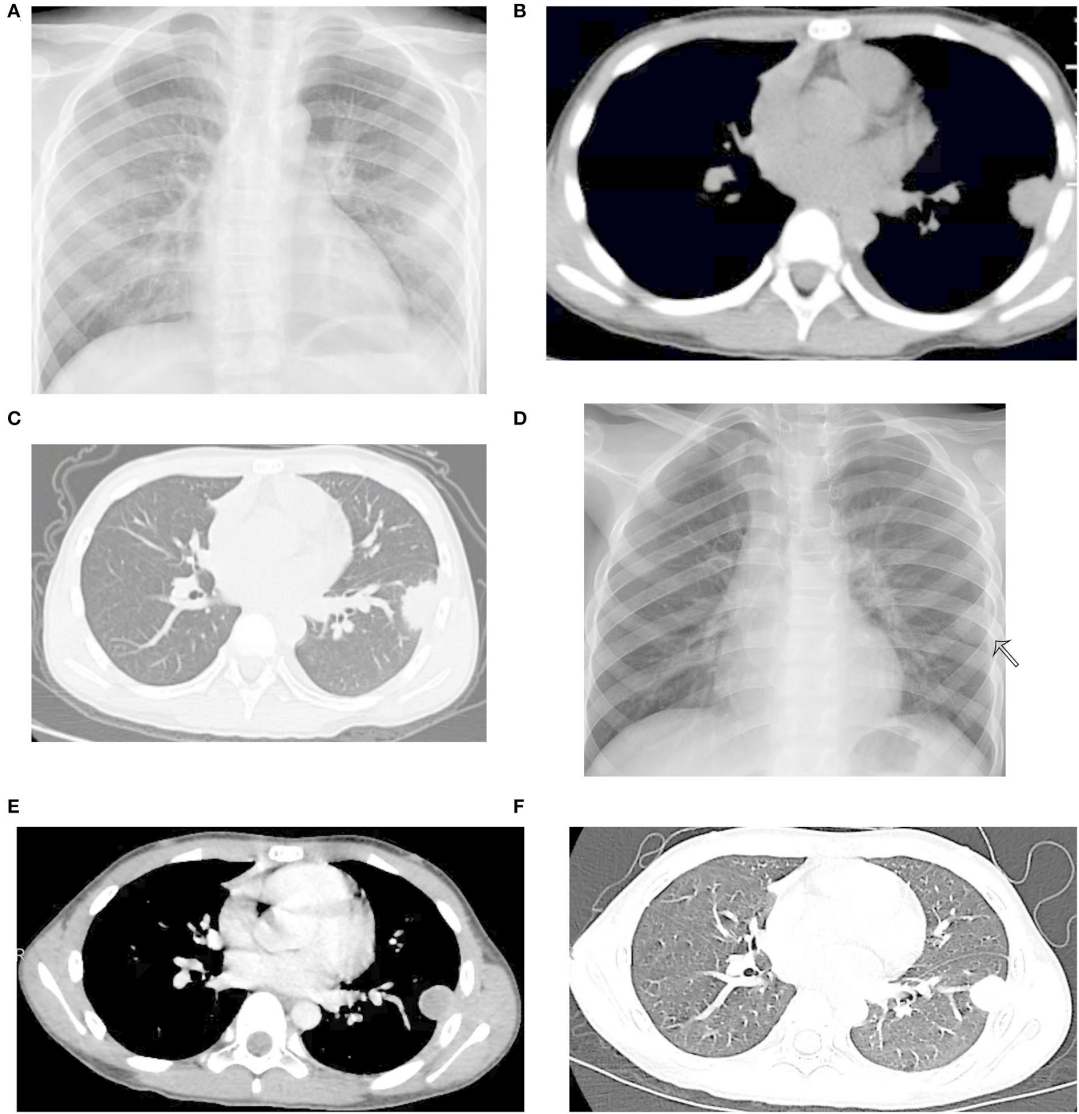
Radiological images before ultransonography (US)-guided lung biopsy. **(A)** Signs of infiltration present in the left lower lung field. **(B,C)** Plane computed tomography (CT) at the previous hospital revealing a round soft tissue mass and a small amount of pleural effusion in the left lung. **(D)** Chest X-ray revealed a left lung nodule measuring 2 cm in diameter. **(E,F)** Contrast-enhanced CT of the chest showing a rim-enhancing lesion in the left lower lobe lung.

A previously healthy 10-year-old boy presented to our department with a 5-day history of intermittent, left-sided chest pain exacerbated by deep breathing. He denied any current fever, cough, hemoptysis, nausea, vomiting, diarrhea, palpitations, night sweats, or weight loss. Additionally, he also denied previous surgery, trauma, sick contacts, dental work, aspiration episodes, and exposure to hazardous and/or infectious materials. He had no remarkable medical history and had received all scheduled vaccines, including BCG, without any serious adverse events. There was no family history of asthma, diabetes, immunodeficiency, malignancy, and tuberculosis. On day 3 of chest pain, he sought treatment at a local hospital. Chest radiography demonstrated left lobe pneumonia (Figure 1A), while blood examination revealed a white blood cell count (WBC) of 10.4× 10^9^/L with 58.5% neutrophils and C-reactive protein levels < 0.50 mg/L. Thus, he was initially treated for community-acquired pneumonia with ceftriaxone and azithromycin for 2 days without relief. A subsequent plane computed tomography (CT) scan of the chest disclosed a round soft tissue mass and a small amount of pleural effusion in the left lung (Figures 1B,C). Then, the patient was transferred to our hospital for further evaluation and treatment at the request of his parents.

**Figure 2:**
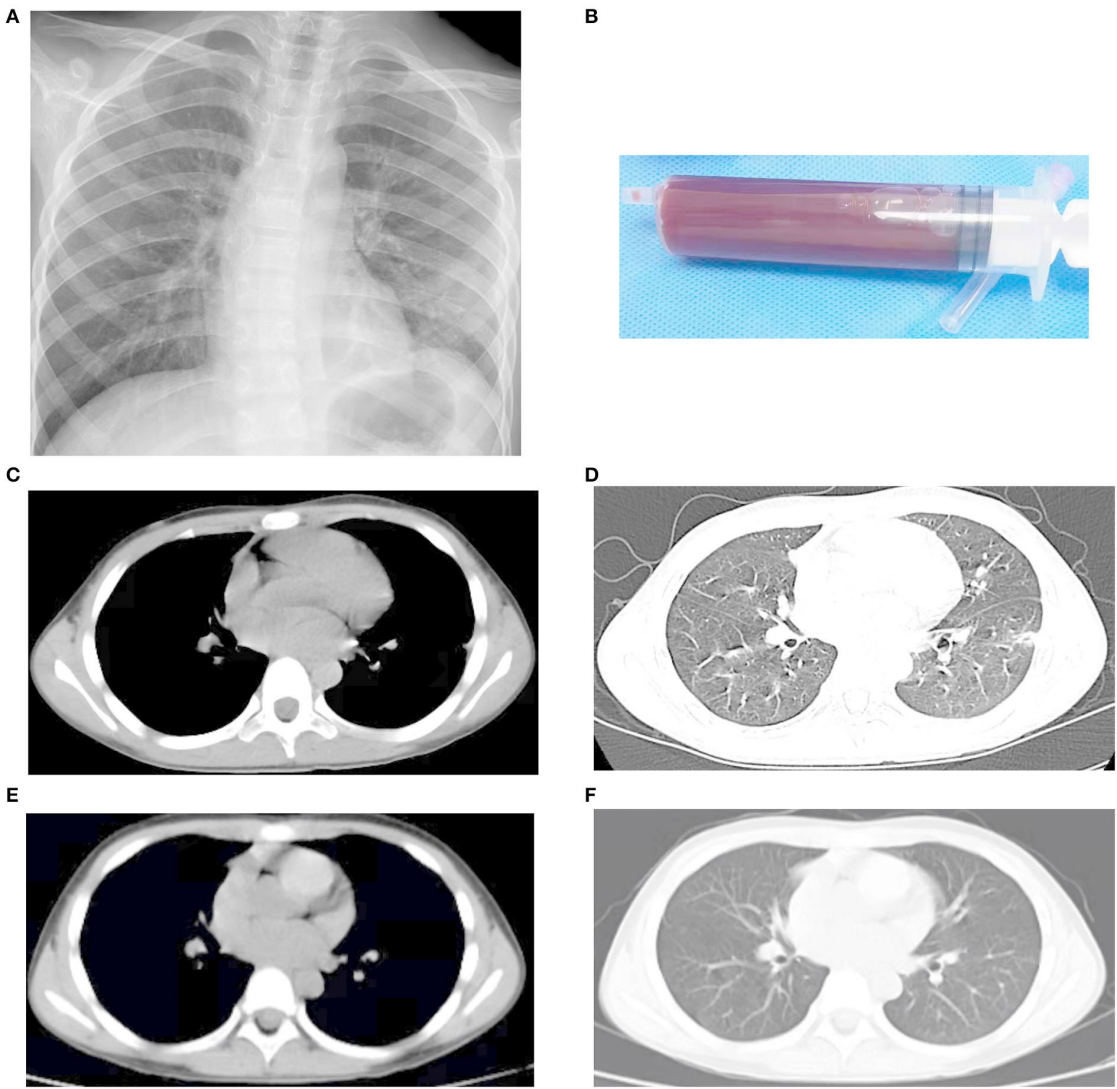
Radiological images after US-guided lung biopsy and appropriate treatment. **(A)** A repeated chest X-ray demonstrating partial resolution of the mass on hospital day 8. **(B)** Reddish-brown purulent pleural fluid. **(C,D)** A repeat chest scan confirming obvious resolution of the lung nodule on hospital day 20. **(E,F)** Follow-up CT after 3 months showing nodule resolution without any residual lesion.

His vital signs upon arriving to us were as follows: body temperature, 37.1°C; pulse rate, 92/min; respiratory rate, 28/min; and blood pressure, 113/67 mmHg. Physical examination showed no abnormalities. Blood routine and biochemical tests were all within normal ranges. Empiric antibiotics with azithromycin and amoxicillin/clavulanate were given. The HIV test, viral hepatitis serologies, antinuclear antibody, serum parasite IgG, serum galactomannan, serum 1, 3-β-D-glucan, serum Cryptococcus antigen test, T-spot test, oncological biomarkers, Legionella antigen, *Mycoplasma pneumoniae* antigen, and respiratory virus antigen were all negative. Due to concern of endocarditis and other occult sources of infection, echocardiogram and abdominal ultrasonography (US) were also ordered and were unremarkable. The patient underwent conventional flexible bronchoscopy and bronchoalveolar lavage (BAL). The utility of bronchoscopy revealed no unsuspected endobronchial lesions. BAL fluid (BALF) yielded only benign bronchial epithelial cells and macrophages, with no bacterial, viral, or fungal inclusions. The initial blood, sputum, and BALF specimens were all negative on both Gram stain and culture. These results yield no clear etiology for the formation of the nodule. However, his symptoms persisted. On hospital day 5, a chest X-ray revealed a left lung nodule measuring 2 cm in diameter (Figure 1D), which was similar to the previous. The thoracic US showed a hypoechoic lesion, 2.2 cm × 1.9 cm × 1.6 cm. This was confirmed by a contrast-enhanced CT scan revealing a rim-enhancing lesion in the left lower lobe lung (Figures 1E,F). The pulmonary abscess was highly suspected based on these findings. He was then sent for a US-guided lung biopsy on hospital day 7. Metagenomic next-generation sequencing (mNGS) showed 416 sequence reads of *S. intermedius* in lung biopsy tissue. Consistently, 5 days later, the culture of the specimen yielded pure growth of *S. intermedius*, with the pathological evaluation of the mass demonstrating fibroblast proliferation, no evidence of malignancy, lymphoma, or soft tissue tumor. The microorganism isolated from the lesion was susceptible to penicillin G, ceftriaxone, vancomycin, linezolid, levofloxacin, and chloramphenicol. Antibiotics were then narrowed to amoxicillin/clavulanate. However, following the biopsy, he had an intermittent slight fever since hospital day 7. A repeated chest X-ray showed partial resolution of the mass (Figure 2A) on hospital day 8, but an ultrasound revealed pleural effusion on hospital day 11. Considering the unsatisfactory effectiveness of amoxicillin/clavulanate, his antibiotic regimen was switched to linezolid on day 11. A thoracentesis was performed evacuating 190 ml of reddish-brown purulent pleural fluid (Figure 2B). Pleural fluid glucose was 6.07 mmol/L, protein 54.4 g/L, lactic dehydrogenase (LDH) 240 U/L, triglyceride 0.42 mmol/L, adenosine deaminase (ADA) 16.2 U/L, and white blood cell count 6,836.0 ×10^6^/L (64.0% polymorphonuclear cells and 36.0% mononuclear cells). Accordingly, pleural fluid analysis with mNGS reported 110 sequence reads of *S. intermedius*. The pleural fluid culture was negative. Clinically, his fever subsided on hospital day 13, and the patient was also much improved. A repeat chest scan confirmed the obvious resolution of the lung nodule and disappearance of the pleural effusion on hospital day 20 (Figures 2C,D). The patient was then discharged home with the plan to finish a 4-week course of linezolid and continued to be asymptomatic. Follow-up CT after 3 months showed nodule resolution without any residual lesion (Figures 2E,F).

## Discussion

Although *S. intermedius* has been recognized as an opportunistic pathogen, it has been implicated in a variety of purulent infections with an apparent tropism for the brain and liver (3). Until now, only a few cases of pneumonia caused by *S. intermedius* have been published in the literature (6–11). The radiological findings shown in the patients with *S. intermedius* pneumonia varied broadly, including consolidation, isolated pleural effusion, lung abscess, and empyema (6–11). Notably, only one case of *S. intermedius* pulmonary nodules in a 29-year-old male was documented (5). Moreover, most of the reported cases were in the adult population (5–11). Therefore, to the best of our knowledge, our case, as a rare phenomenon, is the first ever reported study of isolated pulmonary nodules secondary to *S. intermedius* infection in children. Collectively, these cases highlighted the characteristic property of *S. intermedius* of causing local as well as systemic abscesses (5, 11).

Multiple predisposing conditions were noted related to infection by *S. intermedius*, e.g., poor dental hygiene, chronic sinusitis, immunodeficiency, malignancy, diabetes, previous surgery, and trauma (3). Our case presented a dilemma because it lacked underlying medical problems. Likewise, Esposito et al. reported a 3-year-old girl, without any history of severe congenital or acquired diseases or known risk factors, who developed a 6 × 5.6 cm brain abscess with surgical drainage cultures positive for *S. intermedius* (12). Denby et al. illustrated an unusual case of life-threatening purulent pericarditis secondary to *S. intermedius* in an otherwise healthy male adolescent without typical risk factors (13). Despite the multiple known causes, these data highlight that the source of *S. intermedius* infection can remain unidentified, and *S. intermedius* could infect human subjects regardless of underlying diseases. Although our patient had neither predisposing factors nor a clear source of infection, the possible mechanism for pulmonary nodule formation is aspiration or seeding with transient bacteremia from an oral or gastrointestinal source. Although cell-associated virulence factor (e.g., antigen I/II), extracellular virulence factors (e.g., hyaluronidase, deoxyribonuclease, and chondroitin sulfatase), and intermedilysin contribute to the pathogenic potential of *S. intermedius* (14), the wide range of presentations and radiological findings described in literatures (5–11) raised the question of whether emergent *S. intermedius* species have acquired novel molecular mechanisms of pathogenesis suitable for scientific exploration. Therefore, further identification of disease-specific virulence factors of *S. intermedius* will provide new insight into *S. intermedius* pathogenesis.

Since clinical features of *S. intermedius* infection are often non-specific, it can cause a delay in diagnosis (3, 14, 15). Our patient showed neither fever on admission nor elevated WBC or CRP. Similarly, fever is not present even in severe respiratory disease (e.g., empyema and necrotizing pneumonia) caused by *S. intermedius*, and both WBC and CRP were in the normal range as demonstrated in published cases (9, 11). Therefore, the absence of fever should not dissuade the physician from the seriousness of the infection.

Both X-ray imaging and CT scan showed a nodule of the left lung in our case; however, those imaging modalities were unable to determine the characteristics of the nodule and diagnose definitively. The radiological finding in our case was difficult to distinguish from malignancy, *Cryptococcus neoformans*, and tuberculous nodule, driving us to further perform a US-guided lung biopsy to explore the pathophysiology of the disease. Therefore, a physician should be in high suspicion of *S. intermedius* infection whenever coming across such a similar radiological manifestation. Using US-guided tissue biopsy, we were able to determine the pathology and etiology of the nodule, indicating that the US is a useful technique with low radiation risk for clinical diagnosis and improves the accuracy of the diagnosis. Identification of the causative pathogen is important for appropriate antimicrobial treatment, and susceptibility tests are important. Culture yield and pathological findings with tissue biopsy led us to a final diagnosis, indicating that a tissue biopsy remains to be the best way to definitively diagnose and should be performed if the diagnosis is uncertain. Thus, the approach to determining the etiology of pulmonary nodules should be an overall consideration of clinical presentation, patient history, radiological imaging, laboratory test results, and multidisciplinary collaboration (e.g., US-assisted tissue biopsy) if necessary.

For diagnostic purposes, the diagnosis of *S. intermedius* infection requires isolation of the organism from clinical specimens. Although blood, sputum, and BALF specimen cultures were negative in our case, still, those findings should not exclude the possibility of *S. intermedius* infection. To some extent, the pathogenic role of *S. intermedius* may be underestimated because *S. intermedius* is not commonly discounted as a pathogen but a contaminant using sputum cultivation (16). Additionally, *S. intermedius* is more frequently reported in studies using specimens obtained from transthoracic needle aspiration, intracerebral aspiration, thoracentesis, or percutaneous lung needle aspiration (9, 16–18). Our study and these studies indicate that further lung biopsy and pleural fluid analysis should be performed if *S. intermedius* infection is highly suspected. Physicians should be alert to *S. intermedius* isolated from usual sterile sites or from abscesses.

Pathogen identification in bacterial infections is of great importance for establishing the correct diagnosis and appropriate selection of more targeted treatment. Reported laboratory testing for *S. intermedius* includes culture and 16S rRNA gene sequencing (10, 17). However, culture is time-consuming, and we frequently experience negative culture results, especially for patients who have received antibacterial therapy at the time of specimen sampling. Although 16S rRNA gene sequencing can serve as a useful method for pathogen discovery and identification since the 1990s, it is not used as a routine laboratory test (19). Currently, mNGS, as a revolutionary development for microbiological diagnosis, has been increasingly applied in medical microbiology detection of various diseases, including pneumonia, meningitis, and sepsis (20). It has been proven to be unbiased, culture-independent, high-throughput, and less affected by antibiotic exposure and fast methodology for detecting pathogens, especially for identifying rare, novel, and difficult-to-detect pathogens (21). We revealed the isolated pulmonary nodule secondary to *S. intermedius* infection diagnosed by mNGS. In this way, we showed that mNGS is a valuable supplement to conventional methods for identifying the pathogen responsible for infections with atypical clinical symptoms.

In conclusion, an isolated pulmonary nodule caused by *S. intermedius* infection in an immunocompetent child is an uncommon case with few reports published in the literature. Our case reminds us to be aware of *S. intermedius* infection as a differential diagnosis when physicians encounter a similar pulmonary nodule. In addition, our case report highlights the usefulness of mNGS to identify the primary causative organism of such pulmonary nodules, especially for patients who do not display typical clinical features. Further studies are required to more precisely determine the prevalence and range of clinical features caused by *S. intermedius* infection.

## Data availability statement

The original contributions presented in the study are included in the article/supplementary material, further inquiries can be directed to the corresponding author.

## Ethics statement

Written informed consent was obtained from the individual(s), and minor(s)' legal guardian/next of kin, for the publication of any potentially identifiable images or data included in this article.

## Author contributions

MH and SL collected the data, drafted, edited the manuscript, and contributed equally to this study. XW, DX, and LT revised the manuscript. ZC supervised this study. All authors critically reviewed, revised, approved the final manuscript, and agreed to be responsible for all aspects of this study.

## Funding

This study was supported by a grant from the Zhejiang Provincial Natural Science Foundation (LQ20H190006 and LY21H010002).

## Conflict of interest

The authors declare that the research was conducted in the absence of any commercial or financial relationships that could be construed as a potential conflict of interest.

## Publisher's note

All claims expressed in this article are solely those of the authors and do not necessarily represent those of their affiliated organizations, or those of the publisher, the editors and the reviewers. Any product that may be evaluated in this article, or claim that may be made by its manufacturer, is not guaranteed or endorsed by the publisher.
